# *Helicobacter pylori* infection is correlated with the incidence of erosive oral lichen planus and the alteration of the oral microbiome composition

**DOI:** 10.1186/s12866-021-02188-0

**Published:** 2021-04-20

**Authors:** Shutong Li, Yangheng Zhang, Zongcheng Yang, Jingyuan Li, Ya Li, Huanjie Li, Wenjuan Li, Jihui Jia, Shaohua Ge, Yundong Sun

**Affiliations:** 1 Key Laboratory for Experimental Teratology of Ministry of Education and Department of Microbiology, School of Basic Medical Science, Cheeloo College of Medicine, Shandong University, Jinan, 250012 Shandong China; 2 Department of Periodontology, School and Hospital of Stomatology, Cheeloo College of Medicine, Shandong University & Shandong Key Laboratory of Oral Tissue Regeneration & Shandong Engineering Laboratory for Dental Materials and Oral Tissue Regeneration, Jinan, 250012 Shandong China; 3 Department of Periodontology, Nanjing Stomatological Hospital, Medical School of Nanjing University, 30 Zhongyang Road, Nanjing, 210008 China; 4 Department of Implantology, School and Hospital of Stomatology, Cheeloo College of Medicine, Shandong University & Shandong Key Laboratory of Oral Tissue Regeneration & Shandong Engineering Laboratory for Dental Materials and Oral Tissue Regeneration, Jinan, Shandong People’s Republic of China; 5 School of Medicine, Cheeloo College of Medicine, Shandong University, Jinan, 250012 Shandong China

**Keywords:** *Helicobacter pylori*, Oral lichen planus, Saliva, Microbiome, Inflammatory factor

## Abstract

**Background:**

Oral lichen planus (OLP), a common clinical oral disease, is associated with an increased risk of malignant transformation. The mechanism underlying the pathogenesis of OLP is unknown. Oral dysbacteriosis is reported to be one of the aetiological factors of OLP. Although *Helicobacter pylori* infection is associated with various oral diseases, the correlation between *H. pylori* infection and OLP is unclear. This study aimed to investigate the effect of *H. pylori* infection on OLP pathogenesis and oral microbiome composition in the Chinese population, which has a high incidence of *H. pylori* infection.

**Result:**

In this study, saliva samples of 30 patients with OLP (OLP group) and 21 negative controls (NC group) were collected. *H. pylori* infection was detected using the carbon-13-labeled urea breath test (UBT). The saliva samples were divided into the following four groups based on the *H. pylori* status: *H. pylori*-positive OLP (OLP+), *H. pylori*-positive NC (NC+), *H. pylori*-negative OLP (OLP−), and *H. pylori*-negative NC (NC−). Oral microbiome compositions were significantly different between the OLP and NC groups and between the OLP− and OLP+ groups. Compared with those in the OLP− group, those in the OLP+ group had a higher incidence of erosive OLP and higher levels of salivary cytokines. In contrast, the oral microbiome composition and cytokine levels were not significantly different between the NC− and NC+ groups.

**Conclusions:**

This is the first report to demonstrate that *H. pylori* infection is significantly correlated with the pathogenesis of erosive OLP.

**Supplementary Information:**

The online version contains supplementary material available at 10.1186/s12866-021-02188-0.

## Background

Infection with *Helicobacter pylori* (*H. pylori*), which is classified as a primary carcinogen by the World Health Organization (WHO), can lead to the development of chronic gastritis, gastric ulcer, and gastric cancer [1, 2]. The human oral cavity is a temporary extragastric *H. pylori* reservoir [3]. *H. pylori* has been detected in dental plaques and saliva using various molecular techniques [4, 5]. Additionally, *H. pylori* infection has been associated with oral diseases, such as periodontitis and recurrent oral ulcers [5, 6].

The prevalence of oral lichen planus (OLP), a common chronic inflammatory oral mucosal disease, is 0.5–2.2% in the general adult population [7]. The two main subtypes of OLP are reticular OLP and erosive OLP. Reticular OLP is characterized by protruding white lacy lesions and papules [7, 8], while the erosive OLP is characterized by erythema, erosion, and ulcerative lesions [7, 8]. OLP can cause variable symptoms with different degrees of discomfort, including a prolonged burning sensation, bleeding, tingling, and abnormal taste. Previous studies have demonstrated that OLP, which is defined as a precancerous condition by the WHO, is associated with an increased risk of malignant transformation [9].

Various factors, such as immunological, psychological, infectious, and genetic factors, are reported to be involved in the pathogenesis of OLP [8, 10, 11]. However, the correlation between *H. pylori* infection and OLP has not been elucidated. *H. pylori* has been detected in the periodontal pockets of patients with OLP. Additionally, the presence of *H. pylori* in the oral cavity is associated with leucoplakia and OLP-associated oral lesions [6]. One study detected *H. pylori* nucleic acids in erosive OLP lesions and suggested a possible connection between *H. pylori* and erosive OLP, but they did not perform a statistical analysis [12]. Nevertheless, recent studies have reported that *H. pylori* infection was not detected in the mucosal biopsies of patients with OLP, indicating no correlation between OLP and *H. pylori* infection [13]. Hence, previous studies evaluating the correlation between OLP and *H. pylori* reported contradictory findings. Additionally, most of these studies involved Western populations. In Chinese populations, the average *H. pylori* infection rate is high (more than 50%) [14]. However, the correlation between *H. pylori* and OLP has not been examined in the Chinese population.

Recent studies have demonstrated that the oral microbiome is involved in the development of oral diseases [15]. Oral dysbacteriosis is reported to be one of the aetiological factors of OLP [16]. Indeed, *H. pylori* infection alters the microbiome composition and decreases microbial diversity [17, 18]. One study showed that the eradication of *H. pylori* resulted in changes in the gastric microbiome, including an increase in bacterial diversity [18]. Other studies also found that *H. pylori* infection might be correlated with gut bacteria dysbiosis [19]. Similar results were reported in children with *H. pylori*-induced gastritis [20]. However, the impact of *H.pylori* infection on the oral microbiome still needs further research.

In this study, we aimed to explore the correlation between *H. pylori* infection and OLP. Thus, saliva samples collected from normal control volunteers and patients with OLP were grouped based on *H. pylori* infection status. The clinical subtypes of OLP in *H. pylori*-positive and *H. pylori*-negative patients were determined. The findings of this study indicated that *H. pylori* infection was correlated with an increased incidence of erosive OLP. However, the underlying mechanism was not elucidated. Therefore, the oral microbiome composition of OLP patients with and without *H. pylori* infection was examined to evaluate the effect of *H. pylori* infection on the oral microbiome composition.

## Results

### Patients with OLP had an increased prevalence of *H. pylori* infection

Saliva samples of 30 patients with OLP (OLP group) and 21 normal control volunteers (NC group) were collected to analyse the correlations between *H. pylori* infection status and OLP clinical subtypes. Age and sex were not significantly different between the OLP and NC groups (Table 1).

**Table 1:** Demographic and clinical parameters of the saliva study samples found no statistically significant differences in age and gender of each group

Characteristics	NC-	NC+	OLP-	OLP+	***p value***
	(***n*** = 10)	(***n*** = 11)	(***n*** = 9)	(***n*** = 21)	
*Age (mean* ± *SD)*	46.44 ± 3.772	48.30 ± 3.211	48.30 ± 3.211	46.71 ± 2.618	0.45^a^
*Male/female*	7/3	11/0	8/1	16/5	0.5^b^
*Smoking habit;*	0	0	0	0	
*Alcohol drinking;*	0	0	0	0	
*Antibiotics usage;*	0	0	0	0	
*Systemic disease;*	0	0	0	0	

^a^ANOVA, ^b^Fisher’s test

*H. pylori* infection in the OLP and NC groups was detected using the 13C-UBT. The prevalence of *H. pylori* infection in the OLP group (70%) was markedly higher than that in the NC group (50%) (Table 1).

Erosive OLP was characterized by a red erosive mucosal surface (Fig. S1 in Additional file). Lesion severity and risk of malignant transformation were higher in those with erosive OLP than in those with reticular OLP [9]. In the OLP group, the incidence of erosive OLP in *H. pylori*-positive patients (61.9%) was significantly (chi-square test) higher than that in *H. pylori*-negative patients (11.1%) (*p* = 0.0041). Additionally, *H. pylori* infection was correlated with OLP subtypes (Table 2).

**Table 2:** Chi-sauared test showed statistically significant association of infection of *H. pylori* and the alteration of OLP subtype (*p* < 0.005)

OLP subtype (%)	OLP-	OLP+	***p value***
	(***n*** = 9)	(***n*** = 21)	
*Reticular OLP (n = 16)*	8 (88.9)	8 (38.1)	0.0041^a^
*Erosive OLP (n = 14)*	1 (11.1)	13 (61.9)	

^a^Chi-square test

### Patients with OLP exhibited an altered oral microbiome composition

**Fig. 1:**
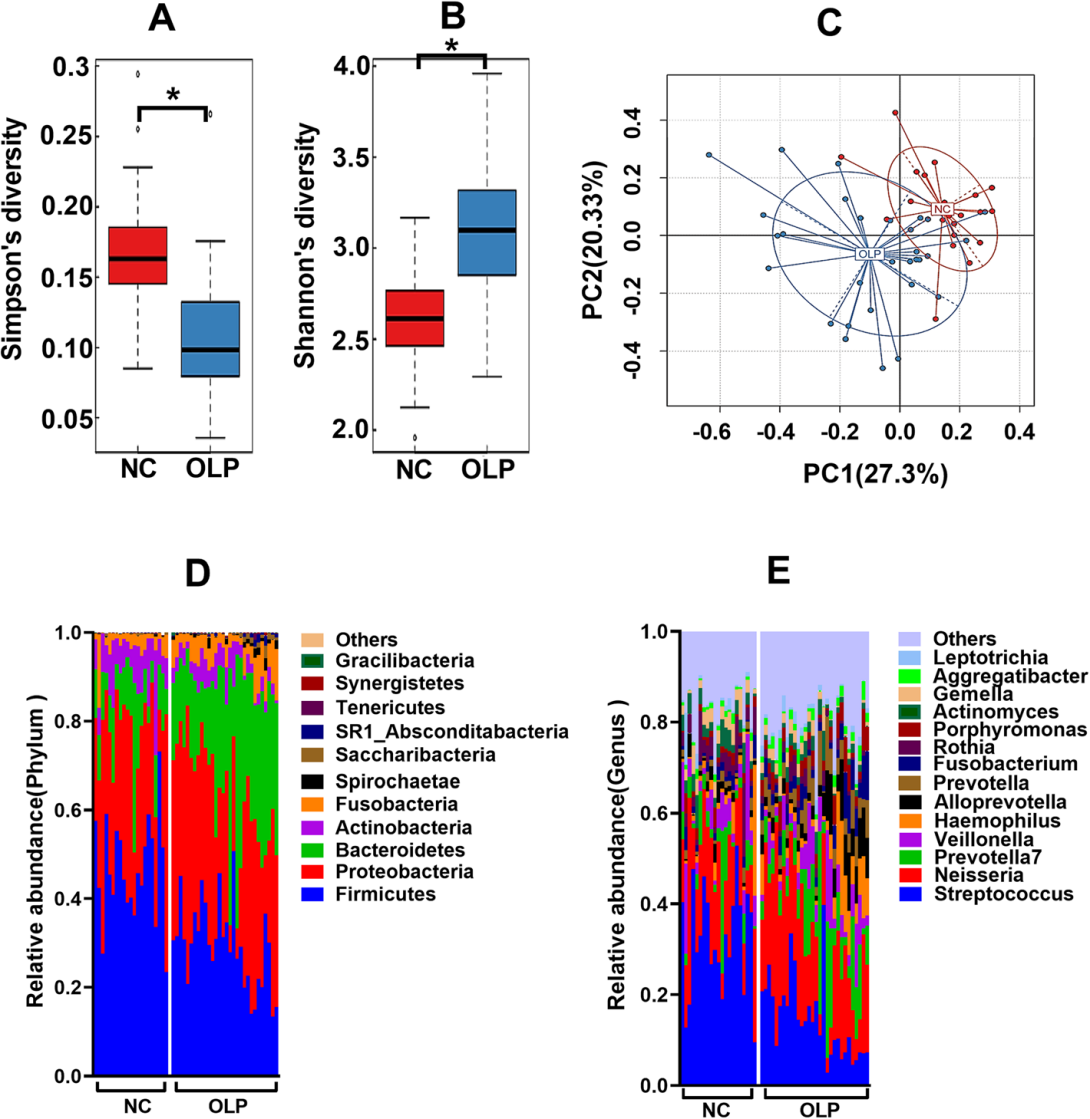
Comparison of the salivary microbiome profiles between the NC (*n* = 21) and OLP groups (*n* = 30). **a** Simpson’s diversity index of salivary microbiome in the NC and OLP groups (*p* = 0.00003). **b** Shannon’s diversity index of salivary microbiome in the NC and OLP groups (*p* = 0.0002). **c** Principal coordinate analysis (PCoA) plots of unweighted UniFrac distances of oral microbiota in the NC and OLP groups. Comparative analysis of the oral microbiome composition at the phylum (**d**) and genus (**e**) levels between the NC and OLP groups. *p < 0.05

Previous studies demonstrated that the oral microbiome composition in patients with OLP was different than that in normal control individuals [21–23]. This study demonstrated that the α diversity of the oral microbiome in the OLP group was significantly higher than that in the NC group (Fig. 1a and b). Principal coordinate analysis (PCoA) revealed that the β diversity of the oral microbiome in the OLP group was significantly different than that in the NC group (Fig. 1c).

Oral microbiome compositions were significantly different between the OLP and NC groups at the phylum (Fig. 1d) and genus levels (Fig. 1e). The dominant oral phyla were *Firmicutes*, *Proteobacteria*, and *Bacteroidetes* (Fig. 1d). Compared with the NC group, the OLP group had a significantly decreased abundance of *Firmicutes* and a significantly increased abundance of *Bacteroidetes* (Fig. 1d). At the genus level, compared with the NC group, the OLP group exhibited a decreased abundance of *Streptococcus* and increased abundances of *Neisseria*, *Prevotella*, and *Prevotella7* (Fig. 1e).

**Fig. 2:**
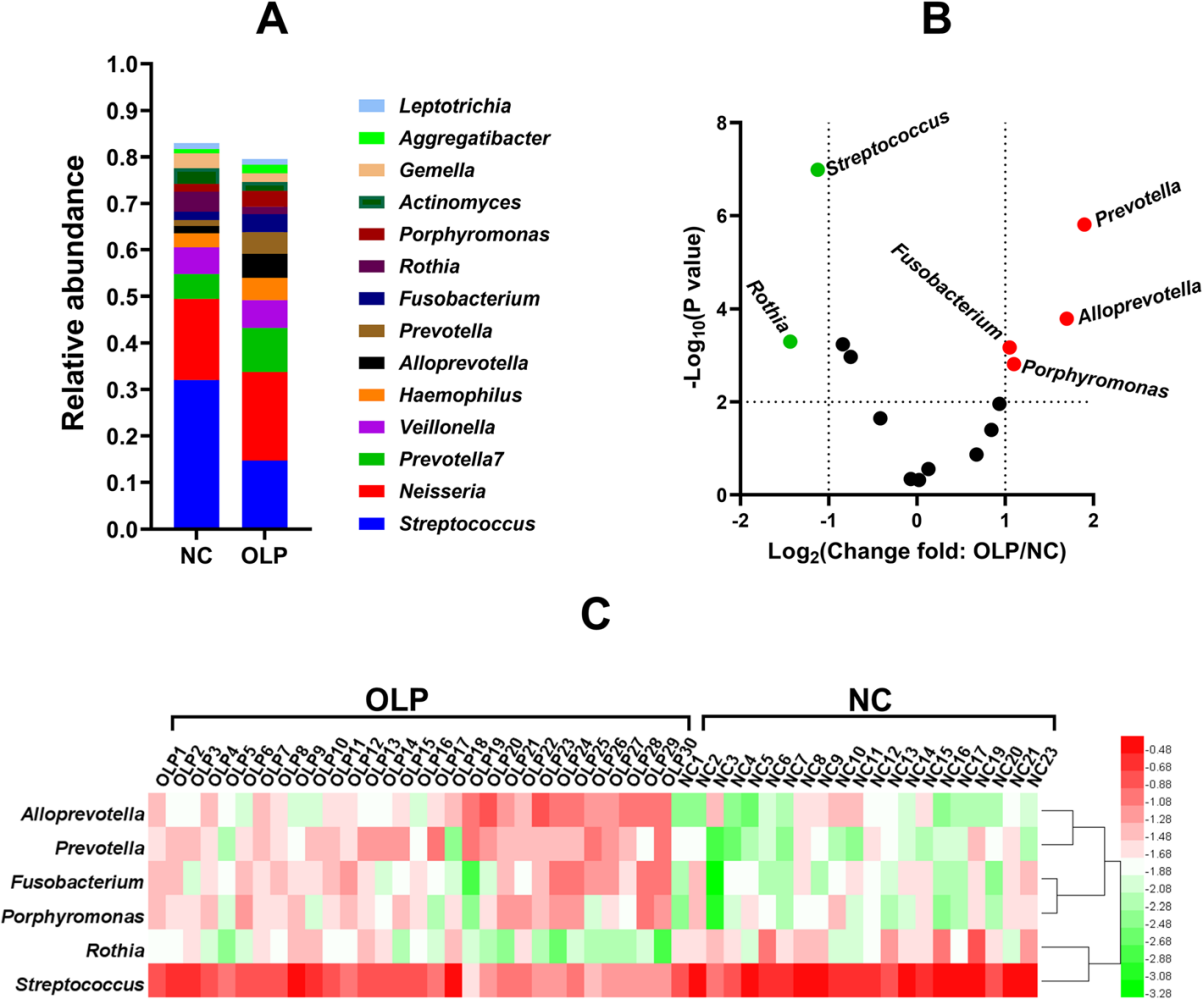
Comparison of the salivary microbiome composition at the genus level between the NC (*n* = 21) and OLP groups (*n* = 30). **a** Comparative analysis of the salivary microbiome composition between the NC and OLP groups (average relative abundance > 1% in the two groups). **b** Volcano plot analysis of salivary microbiome composition between the NC and OLP groups. The *p*-value was calculated using the *t*-test. According to the results shown in panel (**a**), bacteria with a relative abundance greater than 1% were selected and marked as key bacteria. **c** Heat map showing the relative abundances of the key bacteria for sampled individuals of the community subgroups

The bacterial genera with average relative abundances higher than 1% in the oral microbiomes in the OLP and NC groups are listed in Fig. 2a. A volcano plot was used to visalize the differences in the bacterial composition at the genus level between the OLP and NC groups (Fig. 2b). Bacteria with relative abundances greater than 1% and significant differences in relative abundances were screened out (Fig. 2b). Compared with the NC group, the OLP group exhibited decreased relative abundances of *Streptococcus* and *Rothia* and increased relative abundances of *Alloprevotella*, *Prevotella*, *Fusobacterium*, and *Porphyromonas* (Fig. 2b and c).

### *H. pylori* infection alters the salivary microbiome composition in patients with OLP

To analyse the effect of *H. pylori* infection on the salivary microbiome composition in the OLP and NC groups, the saliva samples were divided into the following four groups based on *H. pylori* infection status: OLP+ (*n* = 21); OLP− (*n* = 9); NC+ (*n* = 10); NC− (*n* = 11).

**Fig. 3:**
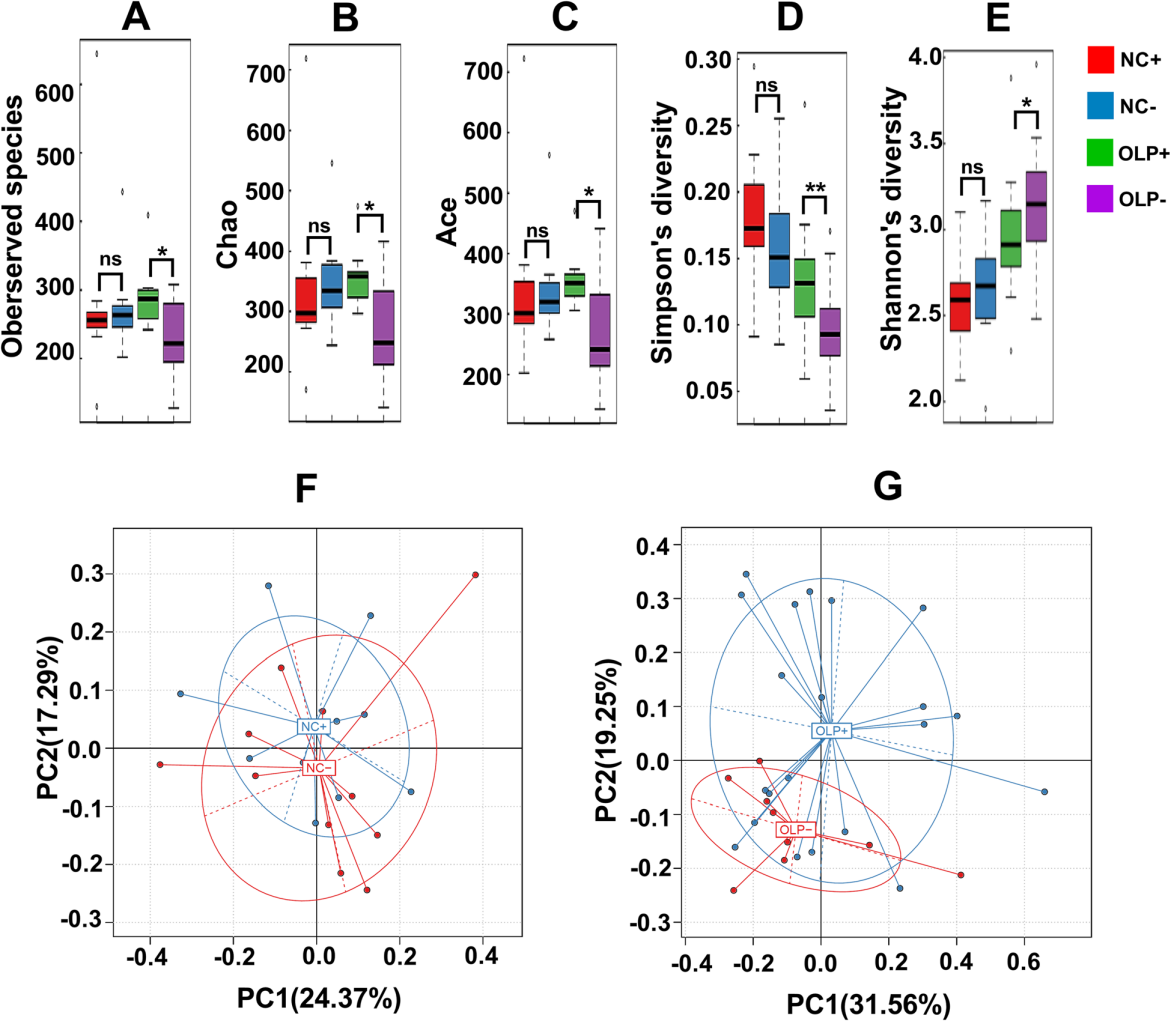
Comparison of the salivary microbiome composition between the NC− (*n* = 11), NC+ (*n* = 10), OLP− (*n* = 9), and OLP + (*n* = 21) groups. **a** Observed species, **b** Chao diversity index, **c** Ace diversity index, **d** Simpson’s diversity index, and (**e**) Shannon’s diversity index between the NC− and NC+ groups, as well as between the OLP− and OLP+ groups. Principal coordinate analysis (PCoA) plots of unweighted UniFrac distances of saliva microbiota between the NC− and NC+ groups (**f**) and between the OLP− and OLP+ groups (**g**). *p < 0.05; **p < 0.01; ns, no significance

The α diversity of the salivary microbiome in the OLP+ group was significantly higher than that in the OLP− group (Fig. 3a, b, c, d and e). In contrast, the α diversity was not significantly different between the NC+ and NC− groups (Fig. 3a, b, c, d and e). PCoA revealed that the β diversity of the salivary microbiome was significantly different between the OLP+ and OLP− groups. However, the β diversity of the salivary microbiome was not significantly different between the NC+ and NC− groups (Fig. 3f and g). Additionally, the bacterial compositions at the genus and phylum levels were not significantly different between the NC+ and NC− groups (Fig. S2 in Additional file).

**Fig. 4:**
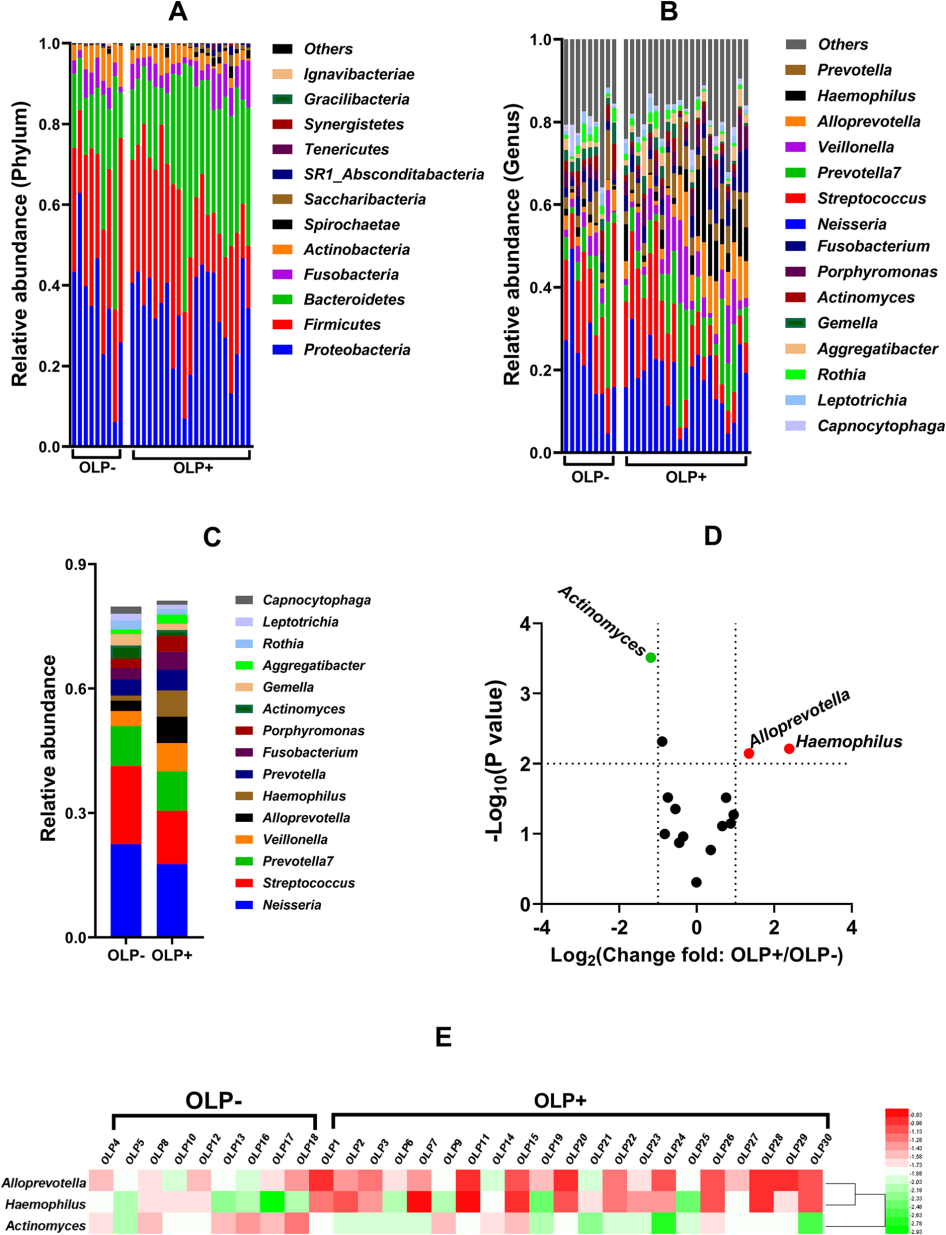
Comparative analysis of the salivary microbiome composition between the OLP− (*n* = 9) and OLP+ (*n* = 21) groups. **a** Comparative analysis of the salivary microbiome composition at the phylum and (**b**) the genus levels between the OLP− and OLP + groups. **c** Comparative analysis of the salivary microbiome composition between the OLP− and OLP+ groups (average relative abundance > 1% in the two groups). **d** Volcano plot analysis of salivary microbiome composition between the OLP− and OLP+ groups. The *p*-value was calculated using the *t*-test. According to the results shown in panel (**c**), bacteria with a relative abundance greater than 1% were selected and marked as key bacteria. **e** Heat map showing the relative abundance of the key bacteria for sampled individuals of the community subgroups

The salivary microbiome compositions were significantly different between the OLP+ and OLP− groups at the phylum and genus levels (Fig. 4a and b). The predominant bacterial phyla were *Proteobacteria*, *Firmicutes*, and *Bacteroidetes* (Fig. 4a), and the relative abundance of *Bacteroidetes* was significantly higher in the OLP+ group than in the OLP− group. At the genus level, the relative abundance of *Alloprevotella* in the OLP+ group was significantly higher than that in the OLP− group (Fig. 4b).

The bacteria of the salivary microbiome in the OLP+ and OLP− groups with an average relative abundances higher than 1% are listed in Fig. 4c. A volcano plot was constructed to visualize the differences in the bacterial composition between the OLP+ and OLP− groups at the genus level (Fig. 4d). Bacteria with a relative abundance higher than 1% and significant differences in the relative abundance were screened out (Fig. 4d). Compared with the OLP− group, the OLP+ group exhibited significantly increased relative abundances of *Alloprevotella* and *Haemophilus* and a significantly decreased relative abundance of *Actinomyces* (Fig. 4d and e).

### Comparative analysis of salivary inflammatory factors

**Fig. 5:**
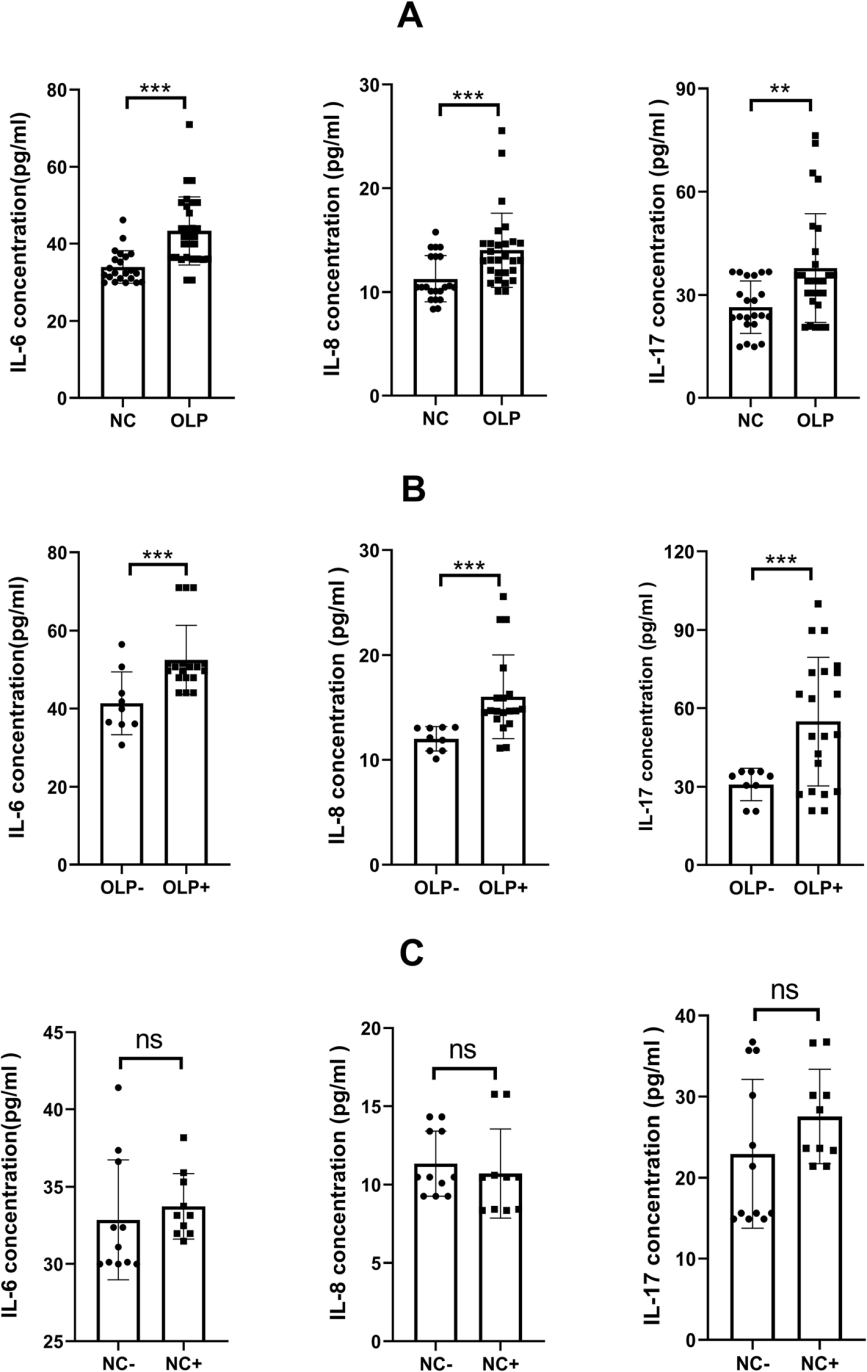
The salivary levels of cytokines (IL-6, IL-8, and IL-17) were measured using ELISA. Comparative analysis of the salivary cytokine levels between the following groups: NC (*n* = 21) and OLP (*n* = 30) groups (**a**); OLP− (*n* = 9) and OLP+ (*n* = 21) groups (**b**); NC− (*n* = 11) and NC+ (*n* = 10) groups (**c**). *p < 0.05; **p < 0.01; ***p < 0.001; ns, no significance

*H. pylori* infection can induce the gastric mucosa to secrete inflammatory factors, such as IL-6, IL-8, and IL-17 [24]. Previous studies have reported the dysregulated expression of various inflammatory factors, such as IL-6, IL-8, IL-17, and TNF-α in patients with OLP [25]. In this study, the salivary levels of IL-6, IL-8, and IL-17 in the OLP and NC groups were analysed using ELISA. The salivary levels of IL-6, IL-8, and IL-17 in the OLP group were significantly higher than those in the NC group (Fig. 5a).

Next, the effect of *H. pylori* infection on the salivary levels of inflammatory factors in the OLP and NC groups was evaluated. Additionally, the salivary levels of IL-6, IL-8, and IL-17 were comparatively analysed between the following groups: the OLP+ and OLP− groups and the NC+ and NC− groups. Compared with those in the OLP− group, the salivary levels of IL-6, IL-8, and IL-17 were significantly increased in the OLP+ group (Fig. 5b). However, the salivary levels of IL-6, IL-8, and IL-17 were not significantly different between the NC+ and NC− groups (Fig. 5c).

**Fig. 6:**
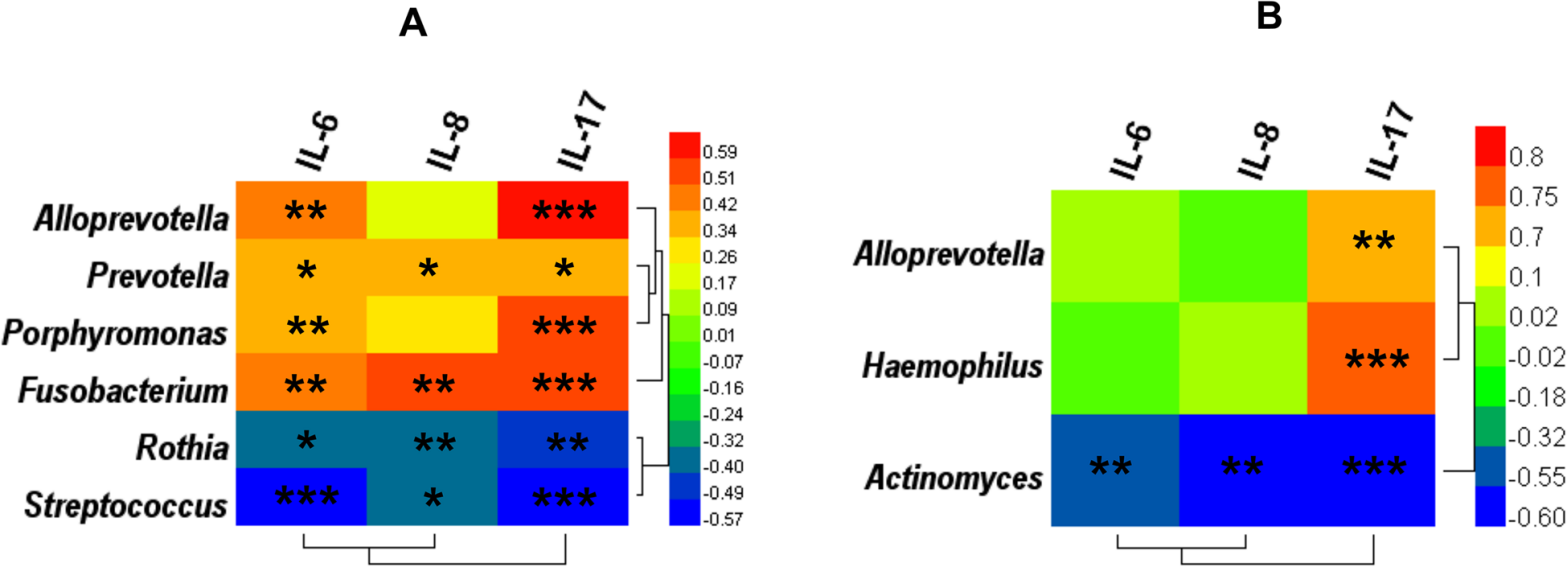
Correlation of cytokine production with the relative abundance of salivary key bacteria at the genus level. **a** NC (*n* = 21) and OLP (*n* = 30) groups; **b** OLP− (*n* = 9) and OLP+ (*n* = 21) groups. The color of cells represents the correlation coefficient (*R*) values. Cells marked with an asterisk show significance after multiple comparisons of Spearman’s correlation, *p < 0.05; **p < 0.01; ***p < 0.001

Next, the correlations between key bacterial genera and inflammatory factors (IL-6, IL-8, and IL-17) were analysed by constructing a heat map of Spearman’s rank correlation coefficients (Fig. 6a and b). In the OLP and NC groups, the abundances of the *Alloprevotella*, *Porphyromonas*, *Fusobacterium*, and *Prevotella* genera were positively correlated with IL-6 and IL-17, while the abundances of the *Prevotella* and *Fusobacterium* genera were positively correlated with IL-8. Furthermore, the abundances of the *Streptococcus* and *Rothia* genera were negatively correlated with IL-7, IL-6, and IL-8 (Fig. 6a). In the OLP+ and OLP− groups, the abundances of the *Alloprevotella* and *Haemophilus* genera were significantly and positively correlated with IL-17, while those of the *Actinomyces* genus were negatively correlated with IL-7, IL-6, and IL-8 (Fig. 6b).

### Correlation of salivary microbiome function with key bacterial genera

**Fig. 7:**
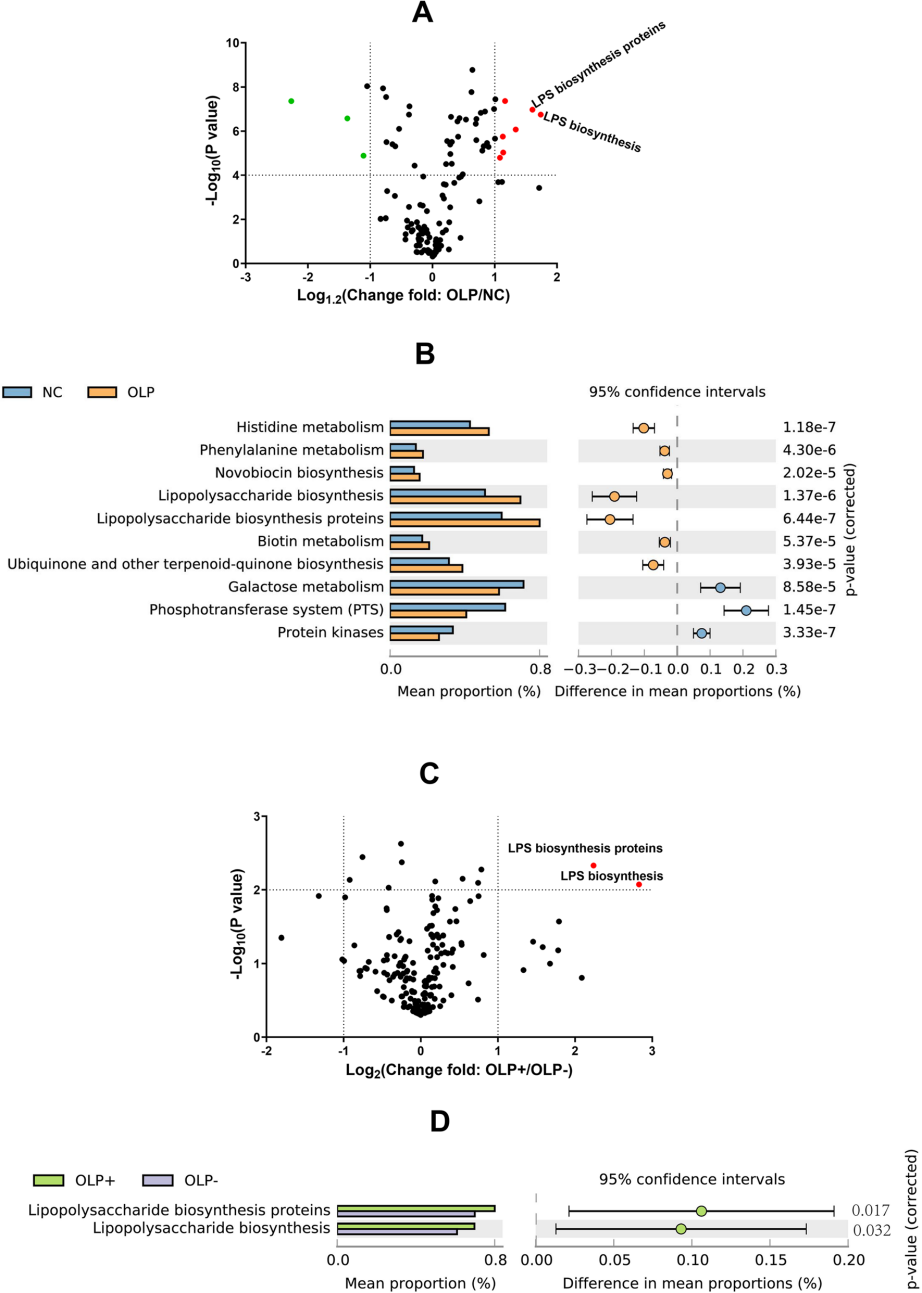
Comparative analysis of the relative abundance of PICRUSt-generated functional profiles of salivary microbiota. Volcano plot analysis of altered Kyoto Encyclopedia of Genes and Genomes (KEGG) pathways between the following groups: NC (*n* = 21) and OLP (*n* = 30) groups (**a**); OLP− (*n* = 9) and OLP+ (*n* = 21) groups (**c**). KEGG pathways with an average relative abundance greater than 2% in the two groups were included. The *p*-value was calculated using the two-tailed paired *t*-test (**b**) and (**d**) based on volcano plot analysis (**a** and **c**). The distinct gene categories were selected according to significant differences in gene categories at level 3 (*t*-test, *p* < 0.05). The bar plots on the left show the mean proportion of each KEGG pathway. The dot plots on the right show the differences in mean proportions between the two indicated groups using *p*-values

PICRUSt was used to predict the metagenome functional content based on 16S rRNA gene sequencing and Kyoto Encyclopedia of Genes and Genomes (KEGG) pathway analysis (Fig. 7). Compared with those in the NC group, the expression levels of genes involved in various metabolic pathways, such as histidine metabolism, phenylalanine metabolism, novobiocin biosynthesis, lipopolysaccharide (LPS) biosynthesis, LPS biosynthesis proteins, biotin metabolism, ubiquinone, and other terpenoid-quinone biosynthesis, in the OLP group were upregulated, while those of genes involved in galactose metabolism, the phosphotransferase system, and protein kinase were downregulated (Fig. 7a and b).

Furthermore, the levels of LPS biosynthesis proteins and LPS biosynthesis in the OLP+ group were higher than those in the OLP− group (Fig. 7c and d).

**Fig. 8:**
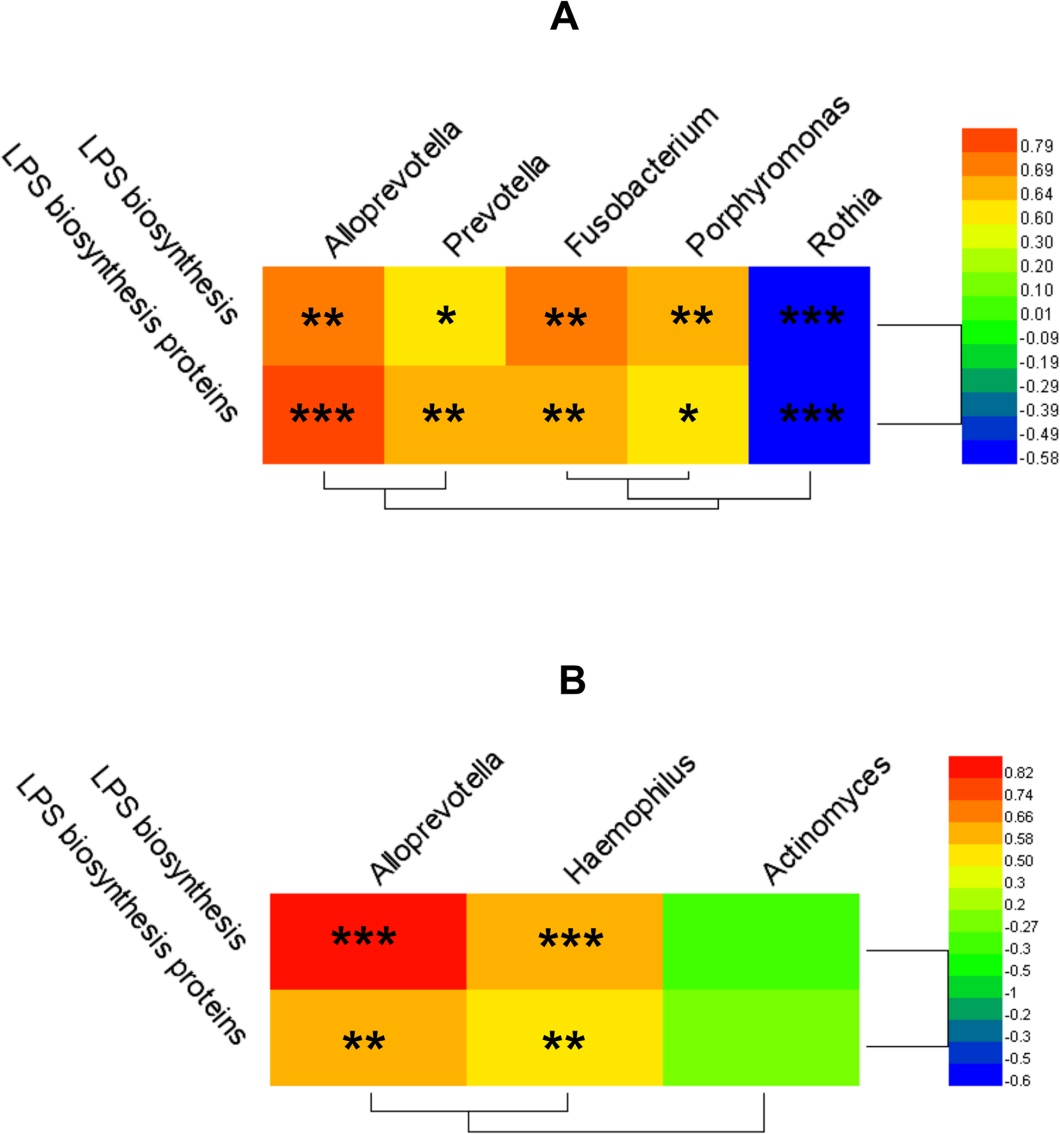
Correlation of PICRUSt-generated key pathway with the relative abundance of salivary key bacteria at the genus level. **a** NC (*n* = 21) and OLP (*n* = 30) groups; **b** OLP− (*n* = 9) and OLP+ (*n* = 21) groups. The color of cells represents the correlation coefficient (*R*) values. Cells marked with an asterisk show significance after multiple comparisons using Spearman’s correlation, *p < 0.05; **p < 0.01; ***p < 0.001

LPS can induce an inflammatory reaction [26]. PICRUSt analysis predicted that the microbial metabolic pathways involved in the pathogenesis of OLP were LPS biosynthesis proteins and LPS biosynthesis. The correlations between key bacterial genera and these two metabolic pathways were examined by constructing a heat map of Spearman’s rank correlation coefficients (Fig. 8a and b). In the OLP and NC groups, the relative abundances of the *Alloprevotella*, *Porphyromonas*, *Fusobacterium*, and *Prevotella* genera were positively correlated, while those of *Rothia* were negatively correlated with the levels of LPS biosynthesis proteins and LPS biosynthesis (Fig. 8a). In the OLP+ and OLP− groups, the abundances of the *Alloprevotella* and *Haemophilus* genera were positively correlated, while those of *Actinomyces* were negatively correlated with the levels of LPS biosynthesis proteins and LPS biosynthesis (Fig. 8b).

## Discussion

This study aimed to analyse the correlation between *H. pylori* infection and the pathogenesis of OLP. The analysis of clinical data indicated that *H. pylori* infection was correlated with an increased incidence of erosive OLP. Patients with OLP exhibited distinct salivary microbiome compositions and increased salivary levels of inflammatory factors. *H. pylori* infection was correlated with an altered composition and altered function of the salivary microbiome, as well as increased salivary levels of inflammatory factors in patients with OLP but not in patients without OLP.

The pathogenesis of OLP, a common clinical oral mucosal disease, is unknown [7, 8]. The proposed causes of OLP include genetic predisposition, cell-mediated hypersensitivity reactions, autoimmune response, stress and infections [8]. Among them, unbalanced cellular-mediated immunity is considered the key pathogenesis of OLP, in which the increased production of T helper 1 (Th1) cytokines is a crucial and early event [7]. Proinflammatory macrophages may exacerbate OLP performance by producing cytokines, such as IL-6, IL-8, and IL-17 [27]. Moreover, oral dysbacteriosis has recently been reported to be one of the aetiological factors of OLP [16]. Studies reported that the oral microbiome of patients with OLP was characterised by increased microbial diversity, decreased *Streptococcus* abundance, and increased *Bacteroides* abundance [28–30].

This study, for the first time, demonstrated that *H. pylori* infection is significantly correlated with the pathogenesis of erosive OLP. One of the main pathogenic mechanisms of *H. pylori* infection is damage to host by inducing local and systemic inflammation, with which Th1 and Th17 cells are associated [2, 31, 32]. IL-6, IL-17 and IL-8 are reported to play an important role in *H. pylori* infection-induced inflammation [31, 33–36]. *H. pylori* infection-induced inflammatory cytokines in the stomach can enter the bloodstream and reach the oral cavity to modulate the immune microenvironment in the oral cavity and aggravate the inflammatory response [37]. In this study, we found that the concentrations of IL-6, IL-8, and IL-17, which are all involved in the pathogenesis of erosive OLP [38], were significantly higher in OLP patients, especially OLP patients with *H. pylori* infection; this may contribute to the pathogenicity of erosive OLP.

The microbiome and the immune microenvironment influence each other [39], and changes in the local immune microenvironment can change the composition of the microbiome [40]. In patients with OLP, *H. pylori* infection may exacerbate the pathological condition of the oral immune microenvironment and disrupt the homeostasis of the salivary microbiome through interactions with different members of the microbial community [41]. This study demonstrated for the first time that *H. pylori* infection is significantly correlated with alterations in the microbiome in OLP patients. Consistent with the findings of previous studies, this study demonstrated that the salivary microbiome composition in patients with OLP was significantly different than that in participants without OLP [21–23]. In this study, OLP patients with *H. pylori* infection exhibited a decreased relative abundance of *Actinomyces* in the salivary microbiome. *Actinomyces* spp., which are core microorganisms in the oral cavity [42], are essential for maintaining the balance of bacterial flora. The decreased abundance of dominant bacteria will lead to the dysregulation of microbiome composition and promote the colonization of other bacteria [43]. In this study, the relative abundances of *Alloprevotella* and *Haemophilus* in the salivary microbiome were significantly higher in the OLP+ group than in the OLP− group. *Haemophilus* spp., gram-negative bacilli, are associated with various opportunistic infections [44–46]. Additionally, *Haemophilus* spp. can activate the macrophages and promote the secretion of proinflammatory cytokines, such as IL-6 and IL-8 [47]. Furthermore, *Haemophilus* spp. infection can induce Th17 cell differentiation and IL-17 secretion and accelerate the recruitment of neutrophils [48].

Next, we analysed the changes in oral microbiome functions caused by *H. pylori* infection. Salivary microorganisms associated with LPS synthesis metabolic pathways were significantly upregulated in OLP patients with *H. pylori* infection and were positively correlated with the relative abundances of *Haemophilus* and *Alloprevotella*. LPS, which exerts immunostimulatory and proinflammatory effects, can promote local inflammatory responses in tissues and upregulate the expression of inflammatory factors [26]. In epithelial cells, LPS binds to TLR4 to promote the secretion of proinflammatory cytokines and chemokines, such as IL-6 and IL-8 [49]. Additionally, LPS can stimulate the secretion of Th17-related cytokines, such as IL-17, in immune cells [50]. Recent studies have demonstrated that LPS inhibits vitamin D receptors in oral keratinocytes, which impairs mucosal homeostasis and leads to epithelial barrier damage. Consequently, this may promote the occurrence and development of erosive OLP [51].

In the NC group, *H. pylori* infection did not enhance the secretion of salivary inflammatory cytokines or alter the microbial community structure. Previous studies have also reported that gastric *H. pylori* infection does not affect the oral microbiome composition [37]. There are conflicting reports on the colonization of *H. pylori* in the oral cavity. The number of *H. pylori* in the oral cavity is low [52]. A meta-analysis revealed that the number of *H. pylori* in saliva was less than that in dental plaque [53]. Chua et al. did not detect *H. pylori* sequences in oral swab samples [37]. In this study, it was difficult to detect *H. pylori* in saliva samples from subjects infected with *H. pylori* through 16S rRNA gene sequencing. In people without OLP, the amount of *H. pylori* in the oral cavity is limited and not enough to induce OLP. However, in patients with OLP, *H. pylori* might aggravate the progression of OLP. Therefore, we speculate that *H. pylori* infection, which is thought to be an aggravating factor rather than an initiating factor, may have no direct effect on the pathogenesis of OLP but may indirectly affect the systemic inflammatory environment and oral microbiome.

The reason for the increased incidence rate of erosive OLP in patients with *H. pylori* infection cannot be fully determined based on the findings of this study. This study elucidated the correlation of *H. pylori* infection with the oral microbiome composition and clinical phenotype of OLP.

## Conclusions

This study elucidated the correlation of *H. pylori* infection with the oral microbiome composition and clinical phenotype of OLP. We demonstrated that *H. pylori* infection was significantly correlated with the pathogenesis of erosive OLP for the first time.

## Methods

### Sample collection

Patients with OLP (OLP group, *n* = 30) who underwent comprehensive clinical and histopathological examinations at the Stomatology Hospital of Shandong University were randomly recruited for this study. The diagnosis of OLP was made by three experienced clinicians and pathologists. According to the clinical classification of OLP by the WHO (2005) [54], the clinical diagnostic criteria are as follows: (1) the presence of white reticular lesions, white papules or plaque-type lesions, with gray-white lines radiating from the papules; (2) presence of a lace-like network of slightly raised gray-white lines; and (3) presence of atrophic lesions with or without erosion and bullae. The histopathologic criteria were as follows: (1) variable epithelial thickness, with saw-tooth rete ridges sometimes observed; (2) the presence of Civatte bodies in the basal layer or superficial lamina propria; (3) the presence of a well-defined band-like zone of lymphocyte infiltration that is confined to the superficial part of the connective tissue; and (4) signs of “liquefaction degeneration” in the basal cell layer. OLP was divided into the following two subtypes: reticular OLP, characterized by white papular lesions, white reticular lesions, or white plaques; and erosive OLP, characterized by erythema and erosive lesions. According to the results of follow-up clinical examinations, age-matched and sex-matched normal control volunteers were recruited (NC group, *n* = 21). All participants’ ages ranged from 18 to 60 years old. The evaluation of sample size was based on ensuring sufficient statistical power to detect whether *H. pylori* infection was related to the OLP subtype as the main result. Considering a significance level of 5% and a power of 80%, according to our calculation, it was determined to be necessary to have 12 individuals for reticular OLP and 12 individuals for erosive OLP respectively. Based on previous research, we identified 51 study subjects [23, 55, 56]. After that, all the study subjects signed informed consent forms. The study subjects did not have a history of periodontitis, dental caries, systemic diseases, smoking, or alcohol consumption. Additionally, the subjects had not undergone antibiotic therapy or received OLP treatment before sample collection. This study was approved by the Medical Ethics Committee of the School of Stomatology of Shandong University (Protocol Number: 20161001).

The saliva samples were collected following the guidelines of the Manual of Procedures for the Human Microbiome Project (http://hmpdacc.org/resources/tools_protocols.php). In brief, unstimulated saliva samples were collected from each patient between 8:00 am and 11:00 am in a sterile DNase/RNase-free conical tube. The study subjects did not consume alcohol or food for at least 2 h before sampling. The samples were transported to the laboratory and stored at − 80 °C until analysis.

*H. pylori* was detected in the OLP and NC groups using a 13C-UBT kit (Headway, Shenzhen, China). According to a previous study, the UBT technique is convenient to use, and it has a sensitivity and specificity above 90% [57]. Based on the *H. pylori* infection status, the samples were divided into the following four groups: *H. pylori*-positive NC (NC+, *n* = 10), *H. pylori*-negative NC (NC−, *n* = 11), *H. pylori*-positive OLP (OLP+, *n* = 21), and *H. pylori*-negative OLP (OLP−, *n* = 9) groups.

### Enzyme-linked immunosorbent assay (ELISA)

The saliva samples (5 mL) were centrifuged at 4 °C and 3500 *g* for 20 min. The supernatant was stored in a DNase/RNase-free EP tube at − 80 °C until further use. The concentrations of IL-6, IL-8, and IL-17 were examined using ELISA kits (Neobioscience Technology Co., Ltd., Shenzhen, China) following the manufacturer’s instructions.

### DNA extraction and 16S rRNA gene amplification

Genomic DNA was isolated from the saliva samples using a QIAamp DNA micro kit (Qiagen, Valencia, CA, USA) following the manufacturer’s instructions. The V3 and V4 regions (336F-806R) of the 16S rRNA gene were amplified using polymerase chain reaction.

### Sequencing and data analysis

High-throughput sequencing was performed at CloudSeq Biotech, Inc. (Shanghai, China). In brief, the raw sequence data were obtained from samples sequenced using an Illumina MiSeq sequencer and subjected to base calling and quality filtering. The samples were separated based on the barcodes. The adaptors were trimmed, and the low-quality reads were removed using Trimmomatic. The paired ends were merged using FLASH. The optimized reads were used for operational taxonomic unit (OTU) clustering, and OTU matrices were generated. The most abundant sequence in each OTU was selected as the representative sequence. The sequences of representative OTUs were compared with those listed in the Greengenes database and those of the samples. Taxonomic abundance matrices were generated. The determination of alpha and beta diversity, as well as statistical analysis and mapping, were performed using Mothur and the R environment. Microbial gene functions were predicted using PICRUSt.

### Statistical analysis

All data are represented as means ± standard deviations. The chi-square test was performed to determine the relationship between *H. pylori* infection and OLP subtype with Statistical Package for Social Science (SPSS) statistical software version 23 (SPSS Inc., Chicago IL, USA). Differences in cytokine levels between two groups were examined using the *t*-tests with GraphPad Prism version 7.01 (GraphPad Software, Inc. CA, USA). The Kruskal-Wallis test was performed to analyse the differences in microbiome compositions between two groups with GraphPad Prism version 9 (GraphPad Software, Inc. CA, USA). The correlation between variables was examined using Spearman’s correlation test with Statistical Package for Social Science (SPSS) statistical software version 23 (SPSS Inc., Chicago IL, USA). A *p-value* below 0.05 was considered significant. *p < 0.05; **p < 0.01; ***p < 0.001.

## Supplementary Information

**Additional file 1.**

## Data Availability

The sequencing data generated in this study are submitted to the Sequence Read Archive of the National Center for Biotechnology Information database (accession number: SRP133987). All data supporting the findings of this study are available in the manuscript. Supplementary material can be obtained from the corresponding author upon reasonable request.
