# Supplementary material for: Helicobacter pylori infection is correlated with the incidence of erosive oral lichen planus and the alteration of the oral microbiome composition
Source: BMC Microbiol. 2021 Apr 20;21:122. doi: 10.1186/s12866-021-02188-0 (PMC8059323; doi:10.1186/s12866-021-02188-0)
Supplement: Supplementary file 1 — Additional file 1. [file 12866_2021_2188_MOESM1_ESM.docx]

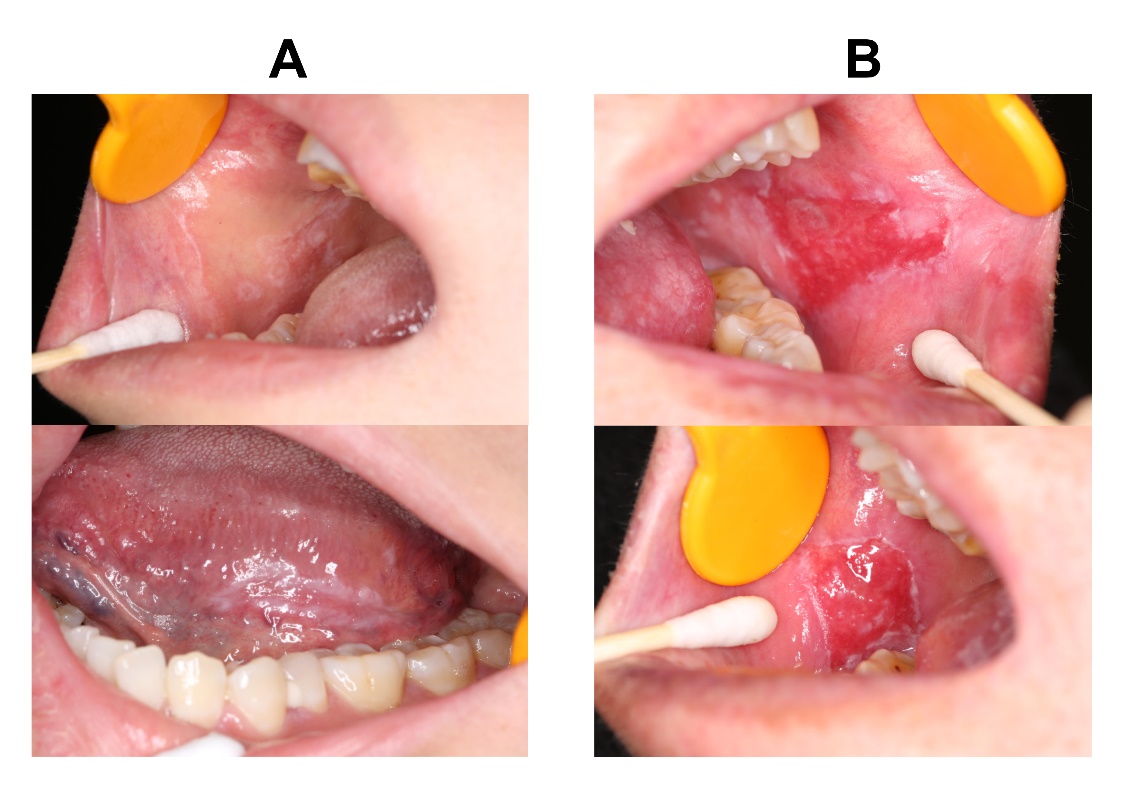


Fig.S1 Clinical subtypes of oral lichen planus (OLP). (A) Reticular OLP, which is the most common form of OLP, is often asymptomatic and appears as multiple papules with networks of raised, white lesions called Wickham striae. (B) Erosive OLP presents as erythematous or atrophic lesions caused by inflammation or epithelial thinning with white striae at the periphery, which results in varying degrees of discomfort, including burning sensation and pain.


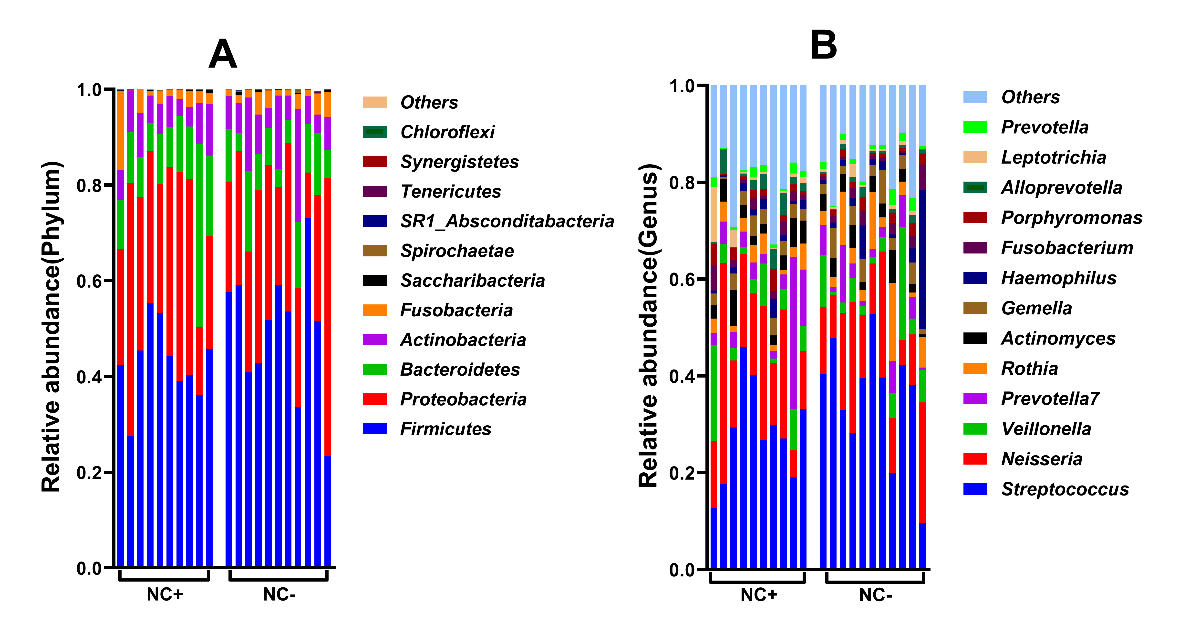


Fig.S2 Comparative analysis of the salivary microbiome composition at the phylum (A) and genus (B) levels between the *H.pylori*-negative normal control (NC−) (n=11) and *H. pylori*-positive normal control (NC+) groups (n=10).
